# Sex Differences in Clinical Outcomes and Surgical Interventions for Infective Endocarditis: A Nationwide Registry

**DOI:** 10.1093/ofid/ofaf473

**Published:** 2025-08-12

**Authors:** Ching-Yi Lin, Feng-Cheng Chang, Chun-Yu Chen, Yu-Ting Cheng, Chia-Pin Lin, Ming-Jer Hsieh, Yi-Hsin Chan, Victor Chien-Chia Wu, An-Hsun Chou, Shao-Wei Chen

**Affiliations:** Department of Medical Education, Chang Gung Memorial Hospital, Linkou Medical Center, Chang Gung University, Taoyuan City, Taiwan; Department of Anesthesiology, Chang Gung Memorial Hospital, Linkou Medical Center, Chang Gung University, Taoyuan City, Taiwan; Department of Anesthesiology, Chang Gung Memorial Hospital, Linkou Medical Center, Chang Gung University, Taoyuan City, Taiwan; Division of Thoracic and Cardiovascular Surgery, Department of Surgery, Chang Gung Memorial Hospital, Linkou Medical Center, Chang Gung University, Taoyuan City, Taiwan; Department of Cardiology, Chang Gung Memorial Hospital, Linkou Medical Center, Chang Gung University, Taoyuan City, Taiwan; Department of Cardiology, Chang Gung Memorial Hospital, Linkou Medical Center, Chang Gung University, Taoyuan City, Taiwan; Department of Cardiology, Chang Gung Memorial Hospital, Linkou Medical Center, Chang Gung University, Taoyuan City, Taiwan; Department of Cardiology, Chang Gung Memorial Hospital, Linkou Medical Center, Chang Gung University, Taoyuan City, Taiwan; Department of Anesthesiology, Chang Gung Memorial Hospital, Linkou Medical Center, Chang Gung University, Taoyuan City, Taiwan; Department of Cardiology, Chang Gung Memorial Hospital, Linkou Medical Center, Chang Gung University, Taoyuan City, Taiwan; Center for Big Data Analytics and Statistics, Chang Gung Memorial Hospital, Linkou Medical Center, Taoyuan City, Taiwan

**Keywords:** epidemiology, infective endocarditis, long-term outcomes, sex differences, valve surgery

## Abstract

**Background:**

Sex differences in infective endocarditis (IE) remain underexplored in large-scale studies. Current findings on clinical outcomes, particularly in surgical IE, are inconsistent, highlighting critical knowledge gaps.

**Methods:**

We conducted a nationwide cohort study using the Taiwan National Health Insurance Research Database (2001–2022) and a total of 28 976 adults with IE were identified. Subgroup analysis focused on 4133 patients undergoing valve surgery. Primary outcomes included in-hospital mortality and long-term survival, analyzed using logistic regression and Cox models.

**Results:**

Of 28 976 patients, 4133 underwent surgery (women/men: 10 580/18 396 overall; 1252/2881 surgical). Women were older with more comorbidities. After propensity score matching, women had higher in-hospital mortality in both the general cohort (21.2% vs 19.8%; odds ratio [OR]: 1.09, 95% confidence interval [CI]: 1.02–1.17) and surgical subgroup (20.7% vs 13.3%; OR: 1.70, 95% CI: 1.37–2.11). Women undergoing surgery had more perioperative complications, including postcardiotomy cardiogenic shock (10.5% vs 7.8%) and de novo dialysis (13.5% vs 9.8%). Long-term mortality was lower in women, while women in the surgical subgroup had higher mortality (hazard ratio [HR]: 1.12, 95% CI: 1.01–1.25) and redo valve surgery rates (11.5% vs 8.1%; HR: 1.43; 95% CI: 1.07–1.90).

**Conclusions:**

Women with IE had higher in-hospital mortality regardless of surgical intervention. Among those who underwent surgery, women experienced more perioperative complications compared with men. Although women exhibited superior overall late survival, those undergoing surgery had worse long-term outcomes. These findings highlight the need for improved sex-specific management, including early diagnosis, timely surgery, and extended follow-up.

## Teaser

- Women with infective endocarditis had higher in-hospital mortality and perioperative complications.
- Women undergoing valve surgery had higher rates of redo valve surgery and long-term mortality.
- Findings highlight the need for sex-specific strategies in managing infective endocarditis.

The significance of infective endocarditis (IE) continues to grow, as reflected by the increase in age-standardized incidence rates from 9.91 to 13.80 per 100 000 person-years between 1990 and 2019, according to the Global Burden of Disease Study [1]. In-hospital mortality rates for IE have remained high over the past 2 decades, ranging from 15% to 30% [2, 3]. Up to 69.3% of confirmed cases meet surgical indications, including heart failure, uncontrolled infection, or embolism risk [2, 4]. Modern challenges, such as an aging population, increased use of implantable cardiac devices, hemodialysis, and rising adult congenital heart disease, contribute to the evolving epidemiology and outcomes of IE [5]. However, critical aspects, such as sex differences, remain poorly understood.

Cardiovascular disease is the leading cause of death among women, accounting for one-third of all deaths [6]. Women-specific risk factors significantly influence the onset and severity of cardiovascular disease but remain under-recognized. Although women constitute a smaller proportion of patients with IE than men [7, 8], they are generally older, frailer, and less likely to undergo surgical treatment [9]. Women more frequently presented with mitral valve infections, while men had higher rates of previous endocarditis, coronary heart disease, and aortic valve infections [10]. Studies on sex differences in IE outcomes have yielded mixed results, with some reporting higher mortality in women [11, 12], others finding no differences in early or 1-year mortality [13, 14], and 1 noting lower fatality rates in women compared with men [15].

Existing evidence is limited by small sample sizes, single-center designs, a focus on operated patients [10, 16], or a lack of long-term follow-up [17]. Utilizing comprehensive real-world data from the Taiwan National Health Insurance Research Database (NHIRD), our study included complete claims, cause-of-death data, and hospital information [18, 19]. Subgroup analysis on patients with IE undergoing valve surgery revealed sex differences in affected valves, prosthetic materials, and intervention modalities. This study provides a comprehensive overview of sex differences in IE, spanning demographics, surgical details, and in-hospital, and long-term outcomes, with a focus on surgical patients over 2 decades on a national scale.

## METHODS

### Data Source

This population-based cohort study utilized data from the Taiwan NHIRD, derived from the government-operated single-payer National Health Insurance (NHI) program, which has covered over 99% of the population since 1995. The NHIRD includes comprehensive data on major cardiac surgeries, demographics, and in-hospital and long-term outcomes. The NHI reimburses costs for invasive procedures and lifesaving treatments, including valve surgeries, mechanical circulatory support, and hemodialysis, making the NHIRD an ideal resource for analyzing IE with extensive follow-up data. The Health and Welfare Data Science Center (HWDC) maintains the NHIRD and its associated databases, such as the death registry. Further details about the NHI and NHIRD are available elsewhere [18, 19]. The accuracy of diagnostic coding in the NHIRD has been supported by several validation studies conducted previously [20, 21]. This study was approved by the Institutional Review Board of Chang Gung Memorial Hospital (registration number: 202400613B0) with a waiver of informed consent.

### Study Population

Patients aged between 18 and 100 years who were hospitalized for IE (either principal or secondary diagnoses) between 1 January 2001 and 31 December 2022 were identified using International Classification of Diseases, Ninth Revision, Clinical Modification (ICD-9-CM) codes (prior to 31 December 2015) and Tenth Revision (ICD-10-CM) codes (after 1 January 2016). Exclusion criteria included patients with missing demographic information, including unknown sex, age, monthly income, urbanization level of the residence, and accreditation level of the hospital (n = 269, 0.9%). After applying the exclusion criteria, 28 976 patients were eligible for inclusion. Propensity score matching at a 1:1 ratio was performed separately for both sexes in the overall cohort and surgical patients with IE (Supplementary Figure 1). Valve surgeries were categorized as aortic, mitral, or tricuspid valve repair or replacement during same IE hospitalization. For patients with 2 or more admissions for IE during the study period, the first admission was designated as the index admission. The ICD diagnostic codes used in the study are listed in Supplementary Table 1.

To evaluate the accuracy of IE diagnosis, we performed a validation study using the Chang Gung Research Database. In Taiwan, the ICD-9 coding system was used through 2015, and ICD-10 has been fully implemented since 2016. A total of 250 hospitalization records (ie, discharge notes and operation records) with a discharge diagnosis of IE (either primary or secondary) were randomly sampled from 2012 to 2019, with equal representation from the ICD-9 and ICD-10 periods (4 years each). After excluding 19 records with insufficient information in the operation or discharge summaries, 231 cases were available for adjudication. Of these, 221 were determined to be true IE cases, yielding a positive predictive value of 95.7%, indicating high diagnostic accuracy. Two physicians (C.-Y. L. and F.-C. C.) independently reviewed the medical charts, and any disagreement was resolved by consensus following consultation with a senior cardiac surgeon (S.-W. C.).

### Covariates

Covariates in this study included age, risk factors for IE, invasive procedures, monthly income, urbanization level, hospital level of the index IE admission (namely the accreditation level, classified as district hospital, regional hospital, and medical center), substance use (including drug abuse and alcoholism), comorbidities, events of history, details of the IE surgery, and any concomitant cardiac surgeries. Disease-related covariates, including substance use, were identified based on diagnostic records from both outpatient and inpatient claims data. Sex, age, monthly income, and urbanization level were extracted from the Registry for Beneficiaries, which contains enrollment information of insured individuals. Surgery-related variables were identified using the NHI reimbursement codes available in the inpatient claims dataset. Invasive procedures included both dental and nondental procedures in the previous 3 months preceding the index IE admission. Dental procedures were identified from outpatient claim data, while nondental procedures were extracted from both outpatient and inpatient claim data.

Risk factors for IE (previous history of IE, prosthetic cardiac valve or material, rheumatic heart disease, and nonrheumatic valve disease) were identified if they were present in any inpatient records preceding the index IE admission. Comorbidities were identified based on at least 2 outpatient diagnoses or any inpatient diagnoses in the year prior to the index IE admission. The status of chronic dialysis and malignancy was confirmed by linking to the catastrophic illness certificate (CIC) database. Given that patients with a CIC are exempted from all NHI copayments for the treatment of the certified disease, the diagnoses recorded in the CIC database are subject to strict verification. Events of history were captured using the previous inpatient diagnoses, including heart failure, stroke, and major bleeding, with data traceable back to 1997. Surgical details of valve procedures (ie, locations of valve, procedure type, and type of prosthesis for replacement) and additional cardiac surgeries were obtained using Taiwan NHI reimbursement codes from inpatient claims data [22, 23]. Nondental invasive procedures were defined as medical or surgical interventions involving mucosal disruption or entry into sterile body sites, excluding any dental interventions; examples include endoscopy with biopsy, colonoscopy with polypectomy, urinary catheterization, bronchoscopy, and central venous catheter insertion.

### Outcomes

We analyzed both in-hospital (perioperative) and long-term outcomes. The primary outcomes of interest were in-hospital mortality and late mortality (including in-hospital death) during follow-up. Information on the dates and causes of death was retrieved by linking to the Taiwan Death Registry database managed by the HWDC. Secondary outcomes included in-hospital complications and long-term outcomes. In-hospital outcomes included cardiogenic shock requiring mechanical circulatory support, re-exploration for bleeding, new-onset stroke, de novo dialysis, tracheostomy, length of hospital stay, and duration of ventilator use. Late outcomes included hospitalization for heart failure, stroke, readmission for IE or any cause, end-stage renal disease requiring permanent dialysis, major bleeding, and pacemaker implantation.

For patients receiving valve surgery, late composite valve complication, redo valve surgery, and major adverse cardiac and cerebrovascular events (MACCEs) were obtained. Late composite outcomes included valve complications, defined as major bleeding, stroke, or readmission for IE. MACCEs were defined as the occurrence of any of the following: all-cause death, stroke, hospitalization for heart failure, or redo valve surgery during follow-up. The analysis of late outcomes was restricted to those patients who survived to discharge, with an exception of late mortality. For the analysis of each late outcome, patients were followed from the discharge date of the index IE hospitalization until the date of event occurrence, death, or the end of database (31 December 2023), whichever occurred first [24, 25].

### Statistical Analysis

Sex differences in baseline characteristics were assessed using standardized differences (STD), calculated as the difference in proportions divided by the square root of the average variance for binary variables and as the difference in means divided by the pooled standard deviation for continuous variables. An absolute STD <0.1 was considered a negligible difference; values around 0.2, 0.5, and 0.8 are conventionally interpreted as small, medium, and large differences, respectively. The trends in sex distribution for the incidence of IE, valve surgeries, and in-hospital deaths over the study period were assessed using the Cochran–Armitage χ^2^ test. To balance differences in demographic and clinical characteristics between sexes, propensity score matching with a 1:1 ratio was conducted separately for the overall cohort and surgical patients with IE. All baseline covariates (listed in Table 1) were included in a multivariable logistic regression model to calculate the propensity score, except that the follow-up year was replaced by the admission date for the index IE episode. Matching was performed using a greedy nearest neighbor algorithm with a caliper of 0.2.

**Table 1.** Baseline Characteristics of Female and Male Patients With IE in the Whole Cohort and in the Subgroup Undergoing Valve Surgery

	Total				With Valve Surgery			
Variable	Total (n = 28 976)	Women (n = 10 580)	Men (n = 18 396)	STD	Total (n = 4133)	Women (n = 1252)	Men (n = 2881)	STD
Age, y	60.2 ± 18.4	63.5 ± 19.0	58.4 ± 17.9	0.28	53.3 ± 15.6	54.8 ± 17.3	52.7 ± 14.8	0.13
Risk of infective endocarditis								
Previous history of infective endocarditis	366 (1.3)	134 (1.3)	232 (1.3)	<0.01	52 (1.3)	8 (.6)	44 (1.5)	−0.09
Prosthetic cardiac valve or material	2060 (7.1)	851 (8.0)	1209 (6.6)	0.06	257 (6.2)	87 (6.9)	170 (5.9)	0.04
Rheumatic heart disease	2743 (9.5)	1187 (11.2)	1556 (8.5)	0.09	446 (10.8)	155 (12.4)	291 (10.1)	0.07
Nonrheumatic valve disease	1065 (3.7)	469 (4.4)	596 (3.2)	0.06	120 (2.9)	44 (3.5)	76 (2.6)	0.05
Invasive procedures in the prior 3 m								
Invasive dental procedures	3282 (11.3)	1235 (11.7)	2047 (11.1)	0.02	497 (12.0)	171 (13.7)	326 (11.3)	0.07
Nondental procedures	3663 (12.6)	1346 (12.7)	2317 (12.6)	<0.01	508 (12.3)	157 (12.5)	351 (12.2)	0.01
Monthly income, NTD								
Quartile 1	9431 (32.5)	3093 (29.2)	6338 (34.5)	−0.11	1208 (29.2)	353 (28.2)	855 (29.7)	−0.03
Quartile 2	9598 (33.1)	3606 (34.1)	5992 (32.6)	0.03	1295 (31.3)	416 (33.2)	879 (30.5)	0.06
Quartile 3	9947 (34.3)	3881 (36.7)	6066 (33.0)	0.08	1630 (39.4)	483 (38.6)	1147 (39.8)	−0.03
Urbanization level								
Low	4330 (14.9)	1547 (14.6)	2783 (15.1)	−0.01	510 (12.3)	135 (10.8)	375 (13.0)	−0.07
Moderate	9514 (32.8)	3327 (31.4)	6187 (33.6)	−0.05	1288 (31.2)	364 (29.1)	924 (32.1)	−0.07
High	8542 (29.5)	3076 (29.1)	5466 (29.7)	−0.01	1227 (29.7)	383 (30.6)	844 (29.3)	0.03
Very high	6590 (22.7)	2630 (24.9)	3960 (21.5)	0.08	1108 (26.8)	370 (29.6)	738 (25.6)	0.09
Hospital level								
Medical center (teaching hospital)	14 443 (49.8)	5286 (50.0)	9157 (49.8)	<0.01	2869 (69.4)	879 (70.2)	1990 (69.1)	0.02
Regional hospital	9715 (33.5)	3530 (33.4)	6185 (33.6)	−0.01	976 (23.6)	286 (22.8)	690 (24.0)	−0.03
District hospital or clinic	4818 (16.6)	1764 (16.7)	3054 (16.6)	<0.01	288 (7.0)	87 (6.9)	201 (7.0)	<0.01
Substance use								
Drug abuse	2724 (9.4)	462 (4.4)	2262 (12.3)	−0.29	391 (9.5)	67 (5.4)	324 (11.2)	−0.21
Alcohol abuse	1108 (3.8)	91 (0.9)	1017 (5.5)	−0.27	155 (3.8)	11 (0.9)	144 (5.0)	−0.25
Comorbidity								
Hypertension	14 241 (49.1)	5854 (55.3)	8387 (45.6)	0.20	1686 (40.8)	533 (42.6)	1153 (40.0)	0.05
Diabetes mellitus	8903 (30.7)	3776 (35.7)	5127 (27.9)	0.17	1006 (24.3)	331 (26.4)	675 (23.4)	0.07
Chronic kidney disease	9154 (31.6)	3805 (36.0)	5349 (29.1)	0.15	1036 (25.1)	349 (27.9)	687 (23.8)	0.09
Dialysis	3708 (12.8)	1966 (18.6)	1742 (9.5)	0.26	369 (8.9)	178 (14.2)	191 (6.6)	0.25
Coronary artery disease	6883 (23.8)	2660 (25.1)	4223 (23.0)	0.05	933 (22.6)	270 (21.6)	663 (23.0)	−0.03
Atrial fibrillation	3236 (11.2)	1388 (13.1)	1848 (10.0)	0.10	451 (10.9)	162 (12.9)	289 (10.0)	0.09
Peripheral arterial disease	2693 (9.3)	943 (8.9)	1750 (9.5)	−0.02	301 (7.3)	99 (7.9)	202 (7.0)	0.03
Liver cirrhosis	2085 (7.2)	566 (5.3)	1519 (8.3)	−0.12	220 (5.3)	39 (3.1)	181 (6.3)	−0.15
Chronic obstructive pulmonary disease	3351 (11.6)	957 (9.0)	2394 (13.0)	−0.13	313 (7.6)	79 (6.3)	234 (8.1)	−0.07
Coagulopathy	1478 (5.1)	528 (5.0)	950 (5.2)	−0.01	199 (4.8)	68 (5.4)	131 (4.5)	0.04
Malignancy	2694 (9.3)	1174 (11.1)	1520 (8.3)	0.10	234 (5.7)	100 (8.0)	134 (4.7)	0.14
Event of history								
Heart failure hospitalization	5387 (18.6)	2463 (23.3)	2924 (15.9)	0.19	647 (15.7)	240 (19.2)	407 (14.1)	0.14
Stroke	4322 (14.9)	1804 (17.1)	2518 (13.7)	0.09	322 (7.8)	104 (8.3)	218 (7.6)	0.03
Major bleeding	4864 (16.8)	1916 (18.1)	2948 (16.0)	0.06	477 (11.5)	166 (13.3)	311 (10.8)	0.08
Follow-up year	5.5 ± 6.2	5.2 ± 6.1	5.8 ± 6.3	−0.10	6.6 ± 5.9	6.1 ± 6.0	6.8 ± 5.8	−0.12

Data were presented as frequency (percentage) or mean ± standard deviation.

Abbreviations: IE, infective endocarditis; NTD, New Taiwan Dollar; STD, standardized difference.

In-hospital outcomes were compared between women and men in both the overall and surgical cohorts using logistic regression for binary outcomes and linear regression for continuous outcomes. Using the Cox proportional hazard model, we compared sex differences in the risks of time-to-event outcomes. Sex was the only explanatory factor in the regression models mentioned above. To account for potential outcome dependency within the same matched pair, we applied a robust sandwich-type variance estimator in the aforementioned regression analyses. We assessed the key model assumptions for linear, logistic, and Cox regression models—namely, normality, linearity in the logit, and the proportional hazards assumption, respectively—and found that they were generally satisfied (data not shown). A 2-sided *P*-value of <.05 was considered statistically significant. Statistical analyses were conducted using SAS version 9.4 (SAS Institute, Cary, NC).

## RESULTS

### Epidemiology of the Infective Endocarditis Populations Between Sexes in Taiwan

**Figure 1.**
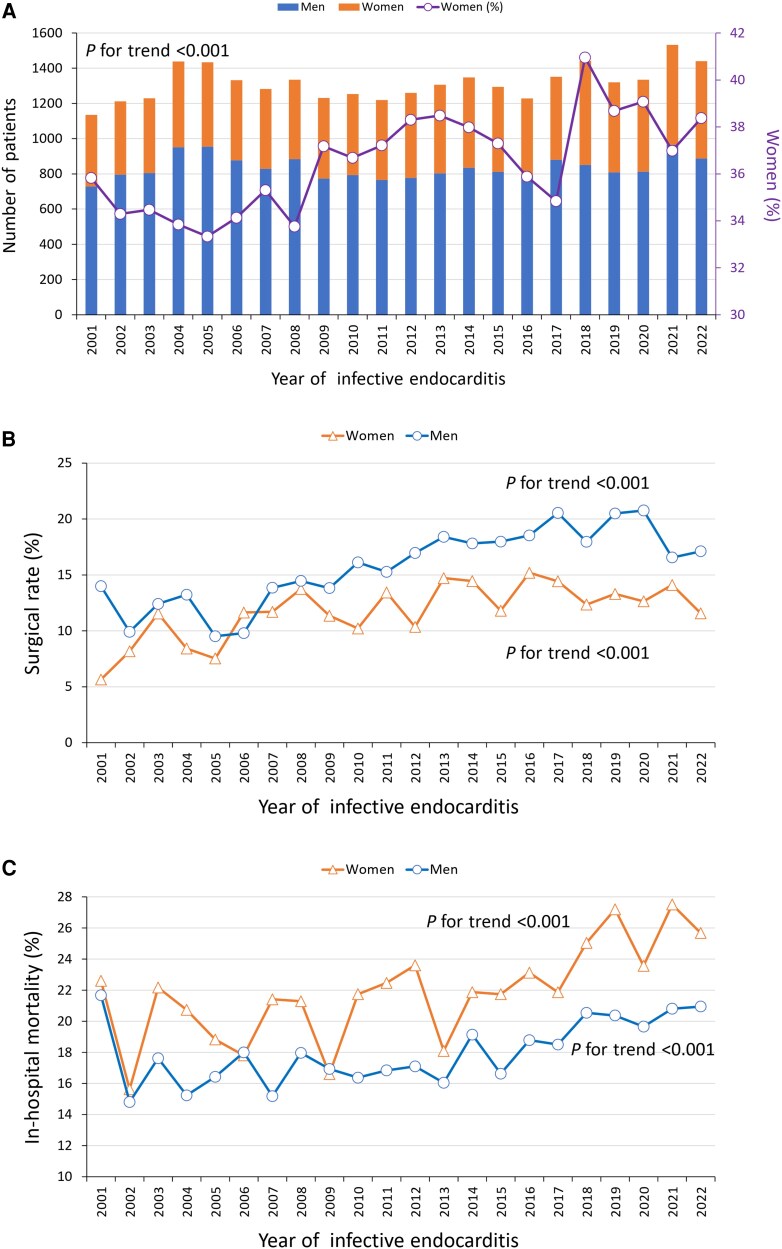
Temporal trends in sex distribution for patients with infective endocarditis (*A*), the proportion of men and women undergoing valve surgeries (*B*), and in-hospital mortality rates (*C*) between 2001 and 2021.

A total of 28 976 patients with IE were identified in this study, with 10 580 women (36.5%) and 18 396 men (63.5%; Supplementary Figure 1). In the operated group, 1252 women (30.3%) and 2881 men (69.7%) were included. The yearly incidence of IE by sex from 2001 to 2022 is depicted in Figure 1*A*, where we noticed a significantly growing trend among women, while men still outnumbered women. To be noticed, the sum of patients with IE remained relatively stable during these 2 decades, fluctuating between 1100 and 1500 each year. Figure 1*B* presents increasing rates of valve surgery performed in both sexes with IE; however, women continuously received surgery less frequently than men (on average, 11.6% vs 17.1%, respectively). During the study period, women consistently had a higher rate of in-hospital death than men and were even statistically growing in temporal trend (Figure 1*C*).

In accordance with the intervened valves for the surgical population during the study period, women with IE underwent mitral valve surgery more commonly than men (Supplementary Figure 2*A*). In contrast, men had aortic valve surgery more often than women (Supplementary Figure 2*B*). Regarding the specific surgical details, the repair rate of mitral valve surgery was comparable between sexes (Supplementary Figure 2*C*), while women received mechanical prosthesis in mitral valve replacement less frequently than men. For aortic valve surgery, the difference in prosthetic selection (bioprosthetic or mechanical) between sexes was not observed (Supplementary Table 2).

### Baseline Characteristics of Patients with Infective Endocarditis

Table 1 presents a comparison of the baseline characteristics of the overall cohort and surgical population between sexes. Women were significantly older, more often comorbid with hypertension, diabetes, chronic kidney disease, dialysis, and heart failure. On the other hand, men were less frequently diagnosed with liver cirrhosis and chronic obstructive pulmonary disease, and more commonly bothered with drug or alcohol abuse. Regarding the associated risk factors for IE and invasive procedures (dental and nondental), sex difference was not observed. There were no significant socioeconomic or hospital-level disparities noted between the sexes. Sex differences in the characteristics of the surgical population were generally consistent with the overall population. After matching, the covariates listed in Table 1 were all well balanced between sexes for both overall and surgical populations. Additionally, details of cardiac surgery for the surgical population were comparable after matching (Supplementary Tables 2 and 3).

### In-hospital Outcomes

Table 2 demonstrates the differences in in-hospital mortality and complications between sexes after matching. Overall, women had a higher risk of in-hospital mortality than men (21.2% vs 19.8%; odds ratio [OR]: 1.09, 95% confidence interval [CI]: 1.02–1.17). Incidence of major complications (eg, newly onset stroke and dialysis) was comparable between sexes. Among the surgical population, women were consistently at higher risk of in-hospital death (20.7% vs 13.3%; OR: 1.70, 95% CI: 1.37–2.11) than men. Concerning perioperative complications, operated women turned out to have more postcardiotomy shock requiring mechanical circulatory support (10.5% vs 7.8%; *P* = .021), more frequently receive de novo dialysis (13.5% vs 9.8%; *P* = .014), and need longer ventilator use. The majority of in-hospital outcomes before matching (Supplementary Table 4) were coherent with the results after matching.

**Table 2.** In-hospital Outcomes of Female and Male Patients With IE in the Whole Cohort and in the Subgroup Undergoing Valve Surgery After Matching

	Total				Valve Surgery			
Variable	Women (n = 9879)	Men (n = 9879)	OR/B (95% CI)	*P*-value	Women (n = 1218)	Men (n = 1218)	OR/B (95% CI)	*P*–value
In-hospital death	2093 (21.2)	1956 (19.8)	1.09 (1.02–1.17)	.016	252 (20.7)	162 (13.3)	1.70 (1.37–2.11)	<.001
30-d mortality	1478 (15.0)	1411 (14.3)	1.06 (.98–1.14)	.177	131 (10.8)	64 (5.3)	2.17 (1.59–2.96)	<.001
Cardiogenic shock requiring MCS	166 (1.7)	151 (1.5)	1.10 (.88–1.38)	.396	128 (10.5)	95 (7.8)	1.39 (1.05–1.83)	.021
Re-exploration for bleeding	38 (0.4)	40 (0.4)	0.95 (.61–1.48)	.821	33 (2.7)	30 (2.5)	1.10 (.67–1.82)	.702
Newly onset stroke	930 (9.4)	887 (9.0)	1.05 (.96–1.16)	.290	176 (14.4)	191 (15.7)	0.91 (.73–1.13)	.396
De novo dialysis	578 (5.9)	566 (5.7)	1.02 (.91–1.15)	.715	165 (13.5)	119 (9.8)	1.45 (1.13–1.86)	.014
Tracheostomy	266 (2.7)	271 (2.7)	0.98 (.83–1.17)	.827	65 (5.3)	66 (5.4)	0.98 (.69–1.40)	.928
Hospital stay, d	32.6 ± 28.6	31.7 ± 26.7	0.85 (.08–1.62)	.031	53.6 ± 32.6	51.7 ± 27.2	1.81 (−.57, 4.20)	.136
Ventilator use, d	4.27 ± 11.21	3.99 ± 10.53	0.28 (−.02, 0.58)	.070	10.15 ± 14.60	8.35 ± 13.34	1.80 (.69–2.91)	.012

Data were presented as frequency (percentage) or mean ± standard deviation.

Abbreviations: *B*, unstandardized regression coefficient; CI, confidence interval; IE, infective endocarditis; OR, odds ratio; MCS, mechanical circulation support.

### Long-Term Mortality and Incidences of Late Outcomes

**Figure 2.**
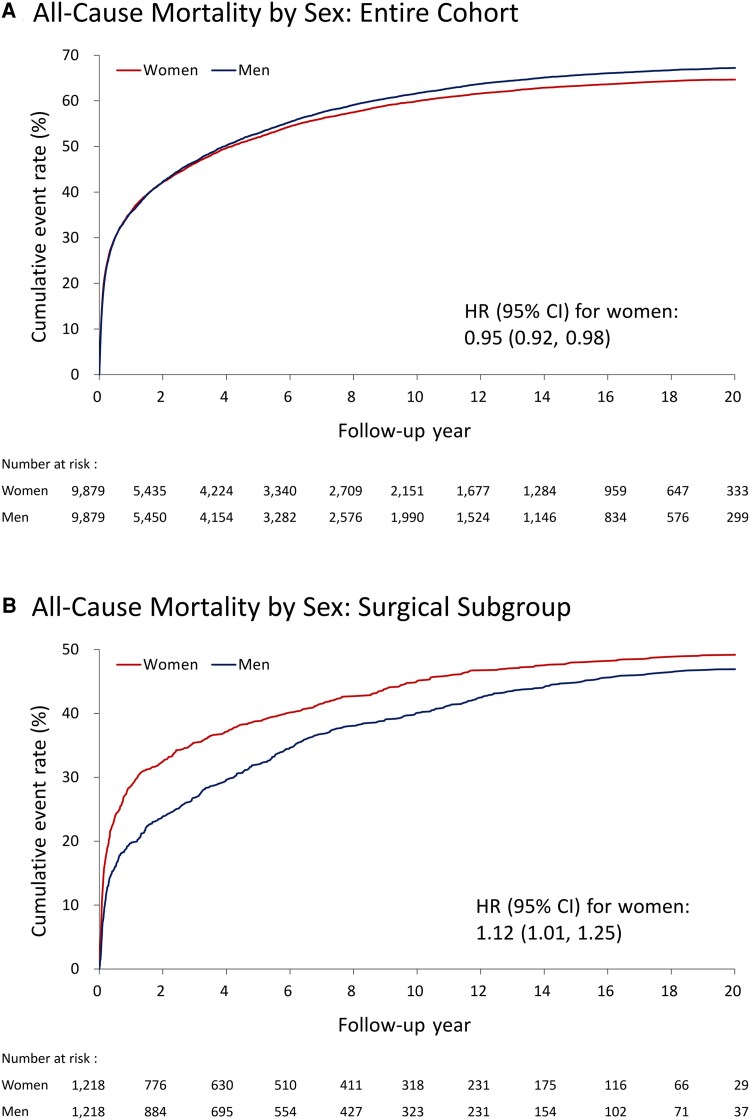
The cumulative event rates of all-cause mortality between men and women in the whole cohort (*A*) and the surgical subgroup (*B*) within the propensity score-matched population. Abbreviations: CI, confidence interval; HR, hazard ratio.

The mean follow-up was 5.5 years in the whole cohort and 6.6 years in the operated subgroup. The cumulative event rates of all-cause mortality (including in-hospital death) were lower in women than in men during follow-up period (64.8% vs 67.5%; hazard ratio [HR]: 0.95; 95% CI: .92–.98; Figure 2*A*). Nevertheless, operated women were observed to had higher long-term mortality compared with men (49.3% vs 47.0%; HR: 1.12; 95% CI: 1.01–1.25; Figure 2*B*). In the matched cohort, the incidences of late adverse events—including hospitalization for heart failure (9.0% vs 8.8%, *P* = .72), end-stage renal disease requiring permanent dialysis (3.5% vs 3.0%, *P* = .18), and pacemaker implantation (3.5% vs 3.1%, *P* = .48)—were comparable between sexes. However, men were more likely to experience stroke (9.7% vs 10.8%; HR for women: 0.86; 95% CI: .78–.95), require re-hospitalization for IE, and suffer from major bleeding (Table 3). Follow-up consequences of the surgical population were consistent with the overall IE population (eg, stroke; 9.9% vs 12.5%; HR for women: 0.77; 95% CI: .59–.99); notably, the incidence of reoperation for valve surgery was higher in operated women (11.5% vs 8.1%; HR: 1.43; 95% CI: 1.07–1.90; Table 3). Supplementary Table 5 listed the late outcomes before matching between the sexes.

**Table 3.** Late Outcomes of Female and Male Patients With IE in the Whole Cohort and in the Subgroup Undergoing Valve Surgery After Matching

	Total				Valve Surgery			
Outcome	Women (n = 7786)	Men (n = 7923)	HR (95% CI)	*P*-value	Women (n = 966)	Men (n = 1056)	HR (95% CI)	*P*-value
Hospitalization for heart failure	697 (9.0)	695 (8.8)	0.98 (.88–1.09)	.724	87 (9.0)	90 (8.5)	1.04 (.77–1.40)	.811
Stroke	758 (9.7)	854 (10.8)	0.86 (.78–.95)	.013	96 (9.9)	132 (12.5)	0.77 (.59–.99)	.044
Readmission due to IE	1270 (16.3)	1472 (18.6)	0.84 (.78–.91)	.001	166 (17.2)	204 (19.3)	0.88 (.72–1.08)	.228
Readmission due to any cause	6278 (80.6)	6388 (80.6)	0.99 (.95–1.02)	.404	714 (73.9)	781 (74.0)	1.02 (.92–1.12)	.737
ESRD requiring permanent dialysis	276 (3.5)	240 (3.0)	1.13 (.95–1.34)	.180	28 (2.9)	19 (1.8)	1.57 (.88–2.82)	.128
Major bleeding	724 (9.3)	909 (11.5)	0.76 (.69–.84)	<.001	86 (8.9)	130 (12.3)	0.69 (.52–.90)	.016
Pacemaker implantation	272 (3.5)	249 (3.1)	1.06 (.90–1.26)	.484	52 (5.4)	54 (5.1)	1.04 (.71–1.51)	.857
Composite valve complication^a^	…	…	…	…	282 (29.2)	346 (32.8)	0.86 (.73–1.01)	.065
Redo valve surgery	…	…	…	…	111 (11.5)	86 (8.1)	1.43 (1.07–1.90)	.015
MACCEs^b^	…	…	…	…	476 (49.3)	534 (50.6)	0.97 (.86–1.09)	.602

Data were presented as frequency (percentage).

Abbreviations: CI, confidence interval; ESRD, end-stage renal disease; HR, hazard ratio; IE, infective endocarditis; MACCEs, major adverse cardiac and cerebrovascular events.

^a^Anyone of major bleeding, stroke, or readmission due to infective endocarditis.

^b^Anyone of all-cause death, stroke, hospitalization for heart failure, or redo valve surgery.

### Sensitivity Analysis

We conducted a sensitivity analysis excluding urbanization level of residence and hospital level of the index IE hospitalization from the propensity score calculation. The results remained highly consistent with the primary analysis for both in-hospital and long-term outcomes (Supplementary Tables 6 and 7).

## DISCUSSION

### Summary of Main Findings

Over the past 2 decades, the incidence of IE has increased among women, who consistently underwent valve surgery at lower rates despite its growing prevalence in both sexes. Women more frequently received mitral valve surgery, while aortic valve interventions were more common in men. In-hospital mortality was higher in women in both the general population and the surgical subgroup, with operated women experiencing more perioperative complications even after propensity score matching. Although no sex differences were observed in overall late survival, women undergoing valve surgery had higher long-term mortality and a greater incidence of redo operations compared with men.

### Sex Difference in Epidemiology of Infective Endocarditis

Consistent with our findings, preceding research found women less frequently diagnosed with IE than men, with a sex ratio around 1:2–1.3 [9, 14, 26–28]. While some attributed this to the higher prevalence of IE risk factors in men, such as prior surgical interventions [11, 29], our data showed no significant sex differences in predisposing factors. In both the United States and our study, women consistently underwent fewer cardiac valve surgeries despite rising surgical rates for both sexes [11]. The higher prevalence of mitral valve IE in women may relate to underlying conditions like mitral valve prolapse and rheumatic disease [30, 31], while aortic valve IE, more common in men, often involves abscesses or fistulas requiring surgery [32, 33]. Women's lower rates of early valve surgery may result from advanced age, increased comorbidities, or mitral valve IE's lower likelihood of causing heart failure [34]. Some even supposed that women more often denied surgery, as they tend to leave against medical advice and seek conservative treatment first [35, 36]. Additionally, women were less likely to receive mechanical mitral valve replacements due to older age and greater intolerance to lifelong anticoagulation [23, 37].

### In-hospital Outcomes of Women and Men

Globally, the in-hospital mortality of patients with IE remains high at 10%–20%, primarily due to comorbidities and complications [4]. The observed sex differences in in-hospital mortality and perioperative complications may, in part, be attributed to baseline differences between sexes. In our cohort, women were older and had a higher burden of comorbidities at presentation, which likely contributed to their increased vulnerability and surgical risks [14]. While propensity score matching mitigates these disparities to some extent, the trend of worse in-hospital outcomes among women persisted. Consistent with our findings, higher in-hospital mortality among women was observed in a Spanish cohort [26], potentially linked to the higher prevalence of *Staphylococcus aureus*, a virulent pathogen more common in women [9, 11, 17]. Delayed medical consultation and postmenopausal estrogen withdrawal, which exacerbates endothelial dysfunction, may also contribute to worse outcomes [35, 38]. A US cohort showed declining in-hospital mortality for both sexes but noted lower surgical rates compared with our study, indicating less severe IE cases lacking surgical indications [11].

Focusing on the surgical group, in-hospital mortality was consistently higher in women than men worldwide [10, 11, 13]. Congestive heart failure, often stemming from late presentation and delayed surgical intervention, remained the leading cause of death in women undergoing valve replacement surgery, particularly for aortic valve IE [11]. Smaller body size in women necessitated the use of smaller prosthetic valves, which has been linked to worse postoperative outcomes [39]. Women also experienced higher rates of postoperative renal dialysis, as reported in a single-center study on aortic valve IE, further predicting poorer survival [16]. A systematic review confirmed consistently higher 30-day mortality in women compared with men across studies [35], with rates reaching 26.7% in a German cohort [10]. The poorer outcomes in this population were likely influenced by a higher prevalence of previous IE and coronary artery disease compared with our cohort.

### Long-Term Outcomes

Consistent with our findings, Stahl et al [9] reported no sex differences in 1- and 5-year mortality from Danish national registries, while Ahtela et al [40] found higher 5- and 10-year mortality in women after adjusting for age and comorbidities in the Finnish registry. Several studies have reported higher 1-year mortality in women compared with men [12, 34, 40]. A Spanish prospective study attributed this to congestive heart failure, the leading cause of death in women, potentially due to lower surgical rates [34]. However, these studies lacked adjustment for confounders like age, comorbidities, and microbiology. Among surgical patients with IE, German researchers observed higher 1-year mortality in women [41], while 2 French cohorts reported similar findings despite long-term benefits of early valve surgery being more pronounced in men [13].

Women may be less tolerant to surgery due to physiological or pathological factors, warranting further investigation. A single-center German study observed no sex-specific differences in crude survival over a median follow-up of 3.9 years, suggesting that the similar or inferior survival rate in women reflects the challenging recovery after IE, outweighing their advantage of longer life expectancy [41]. Although detailed laboratory and imaging data were unavailable in this cohort, prior studies have highlighted sex-specific microbiologic and echocardiographic differences that may contribute to survival disparities after IE [41, 42]. *Staphylococcus aureus*, the most common pathogen in both sexes, has been reported more frequently among operated women in 2 studies, potentially increasing the risk of systemic emboli due to its heightened virulence [43]. While a German surgical cohort found no sex difference in paravalvular abscess or fistula formation, a prospective study of general IE cases suggested fewer intracardiac abscesses in women compared with men [41, 44]. Our findings showed that men with IE were more likely to be hospitalized due to reinfection during follow-up. Prior studies have suggested that men may have higher rates of behavioral risk factors, such as poor dental hygiene, ongoing intravenous drug use, and lower adherence to antibiotic prophylaxis or follow-up care, which could predispose them to recurrent episodes of IE [45, 46]. Notably, our study was the first to report a higher rate of redo valve surgery in women with IE, possibly due to more severe mitral valve damage and longer life expectancy. A French cohort study found most recurrences and late cardiac surgeries occurred within the first year after discharge, with late surgery more common in patients treated medically during the active phase [13]. While surgical rates by sex were not reported, comorbidities, recurrence, and aortic IE were identified as predictors of higher excess mortality in women compared with men [47]. The relationship between virulent pathogens, valve type (native or prosthetic), and infection site with IE recurrence remains unexplored and merits further study.

### Limitations

This study has several limitations due to the inherent constraints of an administrative database. First, it lacks detailed echocardiographic reports, such as those assessing vegetation and heart function. Second, microbiological features related to IE were not available for analysis. Third, information on surgical indications and the timing of surgical interventions is absent. Finally, although propensity score matching was employed to reduce confounding, the possibility of residual confounding from unmeasured or unknown variables cannot be entirely excluded. Despite these limitations, the study has notable strengths. As with all claim-based studies, reliance on ICD codes may introduce misclassification bias; however, the nature of the NHIRD ensures high diagnostic accuracy and reliable identification of complications through comprehensive coding practices. Additionally, the database provides population-wide coverage with long-term follow-up, offering valuable insights into disease outcomes and progression across the entire population.

## CONCLUSIONS

This nationwide study identified marked sex disparities in IE. Women presented at older ages with more comorbidities, underwent surgery less frequently, and experienced higher in-hospital mortality than men. Among surgical patients, women also had higher rates of redo valve surgery and long-term mortality. These findings highlight the need for earlier diagnosis, equitable surgical decision-making, and extended postoperative care strategies for women with IE.

## Supplementary Material

ofaf473_Supplementary_Data

## Supplementary Data

Supplementary materials are available at *Open Forum Infectious Diseases* online. Consisting of data provided by the authors to benefit the reader, the posted materials are not copyedited and are the sole responsibility of the authors, so questions or comments should be addressed to the corresponding author.
