# Supplementary material for: Sex Differences in Clinical Outcomes and Surgical Interventions for Infective Endocarditis: A Nationwide Registry
Source: Open Forum Infect Dis. 2025 Aug 12;12(8):ofaf473. doi: 10.1093/ofid/ofaf473 (PMC12372668; doi:10.1093/ofid/ofaf473)
Supplement: ofaf473_Supplementary_Data [file ofaf473_supplementary_data.zip › Supplementary_Figure_Legends.docx]

**Supplementary Figure 1.** Flowchart illustrates the inclusion and exclusion process for patients in the study.

**Supplementary Figure 2.** The temporal trends in the proportion of men and women undergoing mitral valve surgery (A), aortic valve surgery (B), and mitral valve repair surgery (C) during the study period.

AV, aortic valve; MV, mitral valve.
