# Supplementary material for: Sex Differences in Clinical Outcomes and Surgical Interventions for Infective Endocarditis: A Nationwide Registry
Source: Open Forum Infect Dis. 2025 Aug 12;12(8):ofaf473. doi: 10.1093/ofid/ofaf473 (PMC12372668; doi:10.1093/ofid/ofaf473)
Supplement: ofaf473_Supplementary_Data [file ofaf473_supplementary_data.zip › 20250727 Supplementary Table 1-7.docx]

**Supplementary Table 1.** ICD diagnostic codes used in the study

| Disease | ICD-9 | ICD-10 |
| --- | --- | --- |
| Infective endocarditis | 421.0x, 421.1x, 421.9x | I33, I39 |
| Valve surgery | V422.xx, V433.xx | Z95.2, Z95.3 |
| Rheumatic heart disease | 394.0x, 394.1x, 394.2x, 394.9x, 395.0x, 395.1x, 395.2x, 395.9x, 398.90, 398.91, 398.99 | I05.0, I05.1, I05.2, I05.8, I05.9, I06.0, I06.1, I06.2,I06.8, I06.9, I09.81, I09.89, I09.9 |
| Nonrheumatic valve disease | 394.xx, 395.xx, 396.xx, 397.0x, 397.1x, 397.9x, 424.0x, 424.1x, 424.2x, 424.3x  Excluding: 394.0x, 394.1x, 394.2x, 394.9x, 395.0x, 395.1x, 395.2x, 395.9x, 398.90, 398.91, 398.99 | I07.8, I07.9, I08.0, I08.1, I08.2, I08.3, I08.8, I08.9, I09.1, I34.0, I34.1  Excluding: I05.0, I05.1, I05.2, I05.8, I05.9, I06.0, I06.1, I06.2,I06.8, I06.9, I09.81, I09.89, I09.9 |
| Drug abuse | 304.xx, 305.xx | F11-F19 |
| Alcoholism | V11.3, 291.x, 305.0x, 357.5x, 425.5x, 303.xx, 571.0x, 571.1x, 571.2x, 571.3x, 980.0x | K70, G62.1, I42x6, Z65.8, F10, T51.0X1A, T51.0X2A, T51.0X3A, T51.0X4A |
| Hypertension | 401.xx-405.xx | I10-I15, N26.2 |
| Diabetes mellitus | 250.xx | E08-E13 |
| Chronic kidney disease | 580.xx–589.xx, 403.xx–404.xx, 016.0x, 095.4x, 236.9x, 250.4x, 274.1x, 442.1x, 447.3x, 440.1x, 572.4x, 642.1x, 646.2x, 753.1x, 283.11, 403.01, 404.02, 446.21 | A18.11, E10.2, E11.2, E13.2, I12.0, I13.0, K76.7, M10.3, M31.0, N00.0, N01.0, N02.0, N03.0, N04.0, N05.0, N06.0, N07.0, N08.0, N14.0, N15.0, N15.8, N15.9, N16.0, N17.1, N17.2, N18.0, N19.0, N20.0, N25.0, N26.1, N26.9, N27.0, Q61.0 |
| Dialysis | 585.xx | N18 |
| Coronary artery disease | 410.xx–414.xx | I20-I24 |
| Atrial fibrillation | 427.3x | I48 |
| Peripheral arterial disease | 093.0x, 440.x, 441.x, 443.x, 444.0x, 444.8x, 447.8x, 447.9x, 437.3x, 444.22, 447.1x, 557.1x, 557.9x, V43.4x | I70.0, I71.0, I73.0, I75.0, I77.1, I77.3, I77.89, I79.0, I79.1, I79.2, I79.8, K55.1, K55.8, K55.9, I74.0, I74.3, I74.4, I74.5, I74.8, Z95.8, Z95.9 |
| Liver cirrhosis | 571.2x, 571.5x, 571.6x | K70.2, K70.3, K74.1, K74.2, K74.3, K74.4, K74.5, K74.6 |
| Chronic obstructive pulmonary disease | 491.x, 492.x, 496.x | J41-J44 |
| Coagulopathy | 286.0x-286.9x, 287.1xx, 287.3x-287.5x, 289.81-289.82 | D65, D66, D67, D68, D69.1, D69.3-D69.6, D47.3 |
| Malignancy | 140.xx–208.xx | C00-C96 |
| Heart failure | 428.xx | I50 |
| Ischemic stroke | 433.xx–437.xx | G45.0, G45.1, G45.2, G45.4, G45.8, G45.9, G46.0, G46.1, G46.2, G46.3, G46.4, G46.5, G46.6, G46.7, G46.8, I60.0, I61.0, I62.0, I63.6, I63.8, I63.9, I65.0, I65.1, I65.8, I65.9, I66.0, I67.0, I67.1, I67.2, I67.4, I67.5, I67.6, I67.7, I67.9, I68.0, I68.2, I68.8 |
| Hemorrhage stroke | 430.x–432.x | G45.0, G45.1, G45.2, G45.4, G45.8, G45.9, G46.0, G46.1, G46.2, G46.3, G46.4, G46.5, G46.6, G46.7, G46.8, I60.0, I61.0, I62.0, I63.6, I63.8, I63.9, I65.0, I65.1, I65.8, I65.9, I66.0, I67.0, I67.1, I67.2, I67.4, I67.5, I67.6, I67.7, I67.9, I68.0, I68.2, I68.8 |
| Major bleeding | 336.1x, 363.6x, 372.72, 376.32, 377.42, 379.23, 423.0x, 430xx, 431xx, 432.0x, 432.1x, 432.9x, 530.7x, 531.xx, 531.2x, 531.4x, 531.6x, 532.xx, 532.2x, 532.4x, 532.6x, 533.xx, 533.2x, 533.4x, 533.6x, 534xx, 534.2x, 534.4x, 534.6x, 535.01, 535.11, 535.21, 535.31, 535.41, 535.51, 535.61, 535.71, 537.83, 537.84, 562.02, 562.03, 562.12, 562.13, 569.3x, 569.85, 578xx, 593.81, 719.1x, 729.92, 772.5x, 852.0x, 852.2x, 852.4x, 853.0x, 866.01, 866.02, 866.11, 866.12 | G95.1, H05.23, H11.3, H31.3, H47.02, I31.2, I60.0, I61.0, I62.0, K22.6, K25.0, K26.0, K27.0, K28.0, K29.01, K29.21, K29.31, K29.41, K29.51, K29.61, K29.71, K29.81, K29.91, K31.811, K31.82, K52.81, K55.21, K56.60, K57.01, K57.11, K57.13, K57.21, K57.31, K57.33, K57.81, K57.91, K57.93, K92.0, K92.1, K92.2, M25.0, M79.81, N28.0, P54.4, S06.340A, S06.341A, S06.342A, S06.343A, S06.344A, S06.345A, S06.346A, S06.347A, S06.348A, S06.349A, S06.350A, S06.351A, S06.352A, S06.353A, S06.354A, S06.355A, S06.356A, S06.357A, S06.358A, S06.359A, S06.360A, S06.361A, S06.362A, S06.363A, S06.364A, S06.365A, S06.366A, S06.367A, S06.368A, S06.369A, S06.4X0A, S06.4X1A, S06.4X2A, S06.4X3A, S06.4X4A, S06.4X5A, S06.4X6A, S06.4X7A, S06.4X8A, S06.4X9A, S06.5X0A, S06.5X1A, S06.5X2A, S06.5X3A, S06.5X4A, S06.5X5A, S06.5X6A, S06.5X7A, S06.5X8A, S06.5X9A, S06.6X0A, S06.6X1A, S06.6X2A, S06.6X3A, S06.6X4A, S06.6X5A, S06.6X6A, S06.6X7A, S06.6X8A, S06.6X9A, S31.001A, S37.011A, S37.012A, S37.019A, S37.021A, S37.022A, S37.029A, S37.031A, S37.032A, S37.039A, S37.041A, S37.042A, S37.049A,S37.051A,S37.052A,S37.059A |

Abbreviation: ICD, International Classification of Diseases.

**Supplementary Table 2**. Surgical details of female and male patients with IE in the subgroup undergoing valve surgery before and after matching

|  |  | Before matching | | |  | After matching | | |
| --- | --- | --- | --- | --- | --- | --- | --- | --- |
| Variable | Total  (*n* = 3,819) | Women  (*n* = 1,151) | Men  (*n* = 2,668) | STD |  | Women  (*n* = 1,123) | Men  (*n* = 1,123) | STD |
| Aorta valve repair or replacement | 1,690 (44.3) | 410 (35.6) | 1,280 (48.0) | -0.25 |  | 406 (36.2) | 412 (36.7) | -0.01 |
| Aorta valve replacement with mechanical^a^ | 664 (39.3) | 152 (37.1) | 512 (40.0) | -0.06 |  | 150 (36.9) | 143 (34.7) | 0.05 |
| Mitral valve repair or replacement | 2,343 (61.4) | 775 (67.3) | 1,568 (58.8) | 0.18 |  | 753 (67.1) | 741 (66.0) | 0.02 |
| Mitral valve repair^b^ | 399 (17.0) | 117 (15.1) | 282 (18.0) | -0.08 |  | 116 (15.4) | 116 (15.7) | -0.01 |
| Mitral valve replacement^b^ | 1,963 (83.8) | 666 (85.9) | 1,297 (82.7) | 0.09 |  | 644 (85.5) | 630 (85.0) | 0.01 |
| Mitral valve replacement with mechanical^c^ | 729 (31.1) | 212 (27.4) | 517 (33.0) | -0.12 |  | 211 (28.0) | 196 (26.5) | 0.04 |
| Tricuspid valve repair or replacement | 656 (17.2) | 210 (18.2) | 446 (16.7) | 0.04 |  | 202 (18.0) | 202 (18.0) | <0.01 |
| Double valve replacement | 523 (13.7) | 144 (12.5) | 379 (14.2) | -0.05 |  | 142 (12.6) | 137 (12.2) | 0.01 |
| Concomitant surgery |  |  |  |  |  |  |  |  |
| Coronary artery bypass graft | 282 (7.4) | 70 (6.1) | 212 (7.9) | -0.07 |  | 69 (6.1) | 78 (6.9) | -0.03 |
| Aorta surgery | 143 (3.7) | 48 (4.2) | 95 (3.6) | 0.03 |  | 48 (4.3) | 49 (4.4) | <0.01 |

Abbreviation: IE, infective endocarditis; STD, standardized difference;

a: the denominator is the number of aorta valve surgery;

b: the denominator is the number of mitral valve surgery;

c: the denominator is the number of mitral valve replacement’

Data were presented as frequency (percentage).

**Supplementary Table 3.** Baseline characteristics of female and male patients with IE in the whole cohort and in the subgroup undergoing valve surgery after matching

|  | Total | | | |  | Valve surgery | | | |
| --- | --- | --- | --- | --- | --- | --- | --- | --- | --- |
| Variable | Total  (*n* = 18,434) | Women  (*n* = 9,217) | Men  (*n* = 9,217) | STD |  | Total  (*n* = 2,246) | Women  (*n* = 1,123) | Men  (*n* = 1,123) | STD |
| Age, year | 62.7 ± 18.2 | 62.8 ± 19.2 | 62.6 ± 17.2 | 0.01 |  | 54.4 ± 16.3 | 54.3 ± 17.3 | 54.4 ± 15.2 | <0.01 |
| Risk of infective endocarditis |  |  |  |  |  |  |  |  |  |
| Previous history of infective endocarditis | 190 (1.0) | 95 (1.0) | 95 (1.0) | <0.01 |  | 10 (0.4) | 7 (0.6) | 3 (0.3) | 0.05 |
| Prosthetic cardiac valve or material | 1,495 (8.1) | 729 (7.9) | 766 (8.3) | -0.01 |  | 136 (6.1) | 74 (6.6) | 62 (5.5) | 0.04 |
| Rheumatic heart disease | 2,012 (10.9) | 982 (10.7) | 1,030 (11.2) | -0.02 |  | 263 (11.7) | 129 (11.5) | 134 (11.9) | -0.01 |
| Nonrheumatic valve disease | 5,464 (29.6) | 2,717 (29.5) | 2,747 (29.8) | -0.01 |  | 788 (35.1) | 399 (35.5) | 389 (34.6) | 0.02 |
| Invasive procedures in the prior 3 months |  |  |  |  |  |  |  |  |  |
| Invasive dental procedures | 2,249 (12.2) | 1,103 (12.0) | 1,146 (12.4) | -0.01 |  | 324 (14.4) | 153 (13.6) | 171 (15.2) | -0.05 |
| Non-dental procedures | 2,108 (11.4) | 1,048 (11.4) | 1,060 (11.5) | <0.01 |  | 260 (11.6) | 123 (11.0) | 137 (12.2) | -0.04 |
| Monthly income, NTD |  |  |  |  |  |  |  |  |  |
| Quartile 1 | 5,014 (27.2) | 2,480 (26.9) | 2,534 (27.5) | -0.01 |  | 392 (17.5) | 196 (17.5) | 196 (17.5) | <0.01 |
| Quartile 2 | 5,324 (28.9) | 2,679 (29.1) | 2,645 (28.7) | 0.01 |  | 641 (28.5) | 311 (27.7) | 330 (29.4) | -0.04 |
| Quartile 3 | 8,096 (43.9) | 4,058 (44.0) | 4,038 (43.8) | <0.01 |  | 1,213 (54.0) | 616 (54.9) | 597 (53.2) | 0.03 |
| Urbanization level |  |  |  |  |  |  |  |  |  |
| Low | 2,604 (14.1) | 1,314 (14.3) | 1,290 (14.0) | 0.01 |  | 232 (10.3) | 114 (10.2) | 118 (10.5) | -0.01 |
| Moderate | 5,967 (32.4) | 2,987 (32.4) | 2,980 (32.3) | <0.01 |  | 706 (31.4) | 354 (31.5) | 352 (31.3) | <0.01 |
| High | 5,435 (29.5) | 2,694 (29.2) | 2,741 (29.7) | -0.01 |  | 665 (29.6) | 343 (30.5) | 322 (28.7) | 0.04 |
| Very High | 4,428 (24.0) | 2,222 (24.1) | 2,206 (23.9) | <0.01 |  | 643 (28.6) | 312 (27.8) | 331 (29.5) | -0.04 |
| Hospital level |  |  |  |  |  |  |  |  |  |
| Medical center (teaching hospital) | 9,057 (49.1) | 4,533 (49.2) | 4,524 (49.1) | <0.01 |  | 1,565 (69.7) | 782 (69.6) | 783 (69.7) | <0.01 |
| Regional hospital | 7,684 (41.7) | 3,835 (41.6) | 3,849 (41.8) | <0.01 |  | 667 (29.7) | 334 (29.7) | 333 (29.7) | <0.01 |
| District hospital or clinic | 1,693 (9.2) | 849 (9.2) | 844 (9.2) | <0.01 |  | 14 (0.6) | 7 (0.6) | 7 (0.6) | <0.01 |
| Substance use |  |  |  |  |  |  |  |  |  |
| Drug abuse | 782 (4.2) | 364 (3.9) | 418 (4.5) | -0.03 |  | 104 (4.6) | 52 (4.6) | 52 (4.6) | <0.01 |
| Alcohol abuse | 377 (2.0) | 179 (1.9) | 198 (2.1) | -0.01 |  | 50 (2.2) | 23 (2.0) | 27 (2.4) | -0.02 |
| Comorbidity |  |  |  |  |  |  |  |  |  |
| Hypertension | 9,126 (49.5) | 4,538 (49.2) | 4,588 (49.8) | -0.01 |  | 865 (38.5) | 431 (38.4) | 434 (38.6) | -0.01 |
| Diabetes mellitus | 5,630 (30.5) | 2,814 (30.5) | 2,816 (30.6) | <0.01 |  | 506 (22.5) | 257 (22.9) | 249 (22.2) | 0.02 |
| Chronic kidney disease | 7,426 (40.3) | 3,711 (40.3) | 3,715 (40.3) | <0.01 |  | 710 (31.6) | 361 (32.1) | 349 (31.1) | 0.02 |
| Dialysis | 2,712 (14.7) | 1,344 (14.6) | 1,368 (14.8) | -0.01 |  | 278 (12.4) | 138 (12.3) | 140 (12.5) | -0.01 |
| Coronary artery disease | 3,565 (19.3) | 1,787 (19.4) | 1,778 (19.3) | <0.01 |  | 344 (15.3) | 173 (15.4) | 171 (15.2) | <0.01 |
| Atrial fibrillation | 2,074 (11.3) | 1,024 (11.1) | 1,050 (11.4) | -0.01 |  | 256 (11.4) | 123 (11.0) | 133 (11.8) | -0.03 |
| Peripheral arterial disease | 1,392 (7.6) | 712 (7.7) | 680 (7.4) | 0.01 |  | 134 (6.0) | 69 (6.1) | 65 (5.8) | 0.02 |
| Liver cirrhosis | 927 (5.0) | 465 (5.0) | 462 (5.0) | <0.01 |  | 68 (3.0) | 35 (3.1) | 33 (2.9) | 0.01 |
| Chronic obstructive pulmonary disease | 1,674 (9.1) | 836 (9.1) | 838 (9.1) | <0.01 |  | 123 (5.5) | 64 (5.7) | 59 (5.3) | 0.02 |
| Coagulopathy | 602 (3.3) | 299 (3.2) | 303 (3.3) | <0.01 |  | 77 (3.4) | 41 (3.7) | 36 (3.2) | 0.02 |
| Malignancy | 1,830 (9.9) | 897 (9.7) | 933 (10.1) | -0.01 |  | 160 (7.1) | 85 (7.6) | 75 (6.7) | 0.03 |
| Event of history |  |  |  |  |  |  |  |  |  |
| Heart failure hospitalization | 3,574 (19.4) | 1,762 (19.1) | 1,812 (19.7) | -0.01 |  | 359 (16.0) | 180 (16.0) | 179 (15.9) | <0.01 |
| Stroke | 2,755 (14.9) | 1,367 (14.8) | 1,388 (15.1) | -0.01 |  | 163 (7.3) | 83 (7.4) | 80 (7.1) | 0.01 |
| Major bleeding | 4,401 (23.9) | 2,205 (23.9) | 2,196 (23.8) | <0.01 |  | 336 (15.0) | 169 (15.0) | 167 (14.9) | <0.01 |
| Follow up year | 4.8 ± 5.6 | 4.9 ± 5.7 | 4.8 ± 5.5 | 0.02 |  | 5.7 ± 5.5 | 5.5 ± 5.5 | 6.0 ± 5.4 | -0.09 |

Abbreviation: IE, infective endocarditis; STD, standardized difference; NTD, New Taiwan Dollar;

Data were presented as frequency (percentage) or mean ± standard deviation.

**Supplementary Table 4**. In-hospital outcomes of female and male patients with IE in the whole cohort and in the subgroup undergoing valve surgery before matching

|  | Total | | | |  | Valve surgery | | | |
| --- | --- | --- | --- | --- | --- | --- | --- | --- | --- |
| Variable | Women  (*n* = 9,801) | Men  (*n* = 16,811) | OR/*B* (95% CI) | *P* value |  | Women  (*n* = 1,151) | Men  (*n* = 2,668) | OR/B (95% CI) | *P* value |
| In-hospital death | 2,075 (21.2) | 2,936 (17.5) | 1.27 (1.19, 1.35) | <0.001 |  | 243 (21.1) | 317 (11.9) | 1.99 (1.65, 2.39) | <0.001 |
| 30-days mortality | 1,449 (14.8) | 2,135 (12.7) | 1.19 (1.11, 1.28) | <0.001 |  | 118 (10.3) | 139 (5.2) | 2.08 (1.61, 2.68) | <0.001 |
| Cardiogenic shock requiring MCS | 152 (1.6) | 259 (1.5) | 1.01 (0.82, 1.23) | 0.948 |  | 122 (10.6) | 188 (7.0) | 1.56 (1.23, 1.99) | <0.001 |
| Re-exploration for bleeding | 40 (0.4) | 86 (0.5) | 0.80 (0.55, 1.16) | 0.237 |  | 34 (3.0) | 68 (2.5) | 1.16 (0.77, 1.77) | 0.476 |
| Newly-onset stroke | 725 (7.4) | 1,376 (8.2) | 0.90 (0.82, 0.98) | 0.022 |  | 130 (11.3) | 319 (12.0) | 0.94 (0.76, 1.17) | 0.560 |
| De novo dialysis | 529 (5.4) | 952 (5.7) | 0.95 (0.85, 1.06) | 0.362 |  | 159 (13.8) | 290 (10.9) | 1.31 (1.07, 1.62) | 0.010 |
| Tracheostomy | 298 (3.0) | 473 (2.8) | 1.08 (0.94, 1.26) | 0.287 |  | 67 (5.8) | 126 (4.7) | 1.25 (0.92, 1.69) | 0.156 |
| Hospital stay, days | 31.6 ± 28.1 | 31.6 ± 26.3 | 0.04 (-0.63, 0.71) | 0.905 |  | 53.4 ± 31.9 | 50.7 ± 26.9 | 2.67 (0.70, 4.64) | 0.008 |
| Ventilator use, days | 4.1 ± 10.9 | 3.8 ± 10.1 | 0.26 (-0.001, 0.52) | 0.051 |  | 10.4 ± 14.7 | 7.8 ± 12.6 | 2.55 (1.63, 3.46) | <0.001 |

Abbreviation: IE, infective endocarditis; OR, odds ratio; *B*, unstandardized regression coefficient; CI, confidence interval; MCS, mechanical circulation support;

Data were presented as frequency (percentage) or mean ± standard deviation.

**Supplementary Table 5**. Late outcomes of female and male patients with IE in the whole cohort and in the subgroup undergoing valve surgery before matching

|  | Total | | | |  | Valve surgery | | | |
| --- | --- | --- | --- | --- | --- | --- | --- | --- | --- |
| Outcome | Women  (*n* = 7,726) | Men  (*n* = 13,875) | HR (95% CI) | *P* value |  | Women  (*n* = 908) | Men  (*n* = 2,351) | HR (95% CI) | *P* value |
| Hospitalization for heart failure | 697 (9.0) | 1,094 (7.9) | 1.21 (1.10, 1.33) | <0.001 |  | 72 (7.9) | 178 (7.6) | 1.04 (0.79, 1.37) | 0.789 |
| Stroke | 620 (8.0) | 1,230 (8.9) | 0.95 (0.87, 1.05) | 0.343 |  | 63 (6.9) | 233 (9.9) | 0.68 (0.51, 0.90) | 0.006 |
| Readmission due to IE | 1,128 (14.6) | 2,571 (18.5) | 0.79 (0.74, 0.85) | <0.001 |  | 131 (14.4) | 392 (16.7) | 0.86 (0.71, 1.05) | 0.148 |
| Readmission due to any cause | 6,059 (78.4) | 10,751 (77.5) | 1.07 (1.04, 1.11) | <0.001 |  | 635 (69.9) | 1,630 (69.3) | 1.04 (0.95, 1.14) | 0.384 |
| ESRD requiring permanent dialysis | 249 (3.2) | 371 (2.7) | 1.27 (1.08, 1.49) | 0.004 |  | 24 (2.6) | 53 (2.3) | 1.16 (0.72, 1.88) | 0.552 |
| Major bleeding | 714 (9.2) | 1,611 (11.6) | 0.82 (0.75, 0.90) | <0.001 |  | 66 (7.3) | 242 (10.3) | 0.69 (0.52, 0.90) | 0.006 |
| Pacemaker implantation | 259 (3.4) | 356 (2.6) | 1.39 (1.18, 1.63) | <0.001 |  | 44 (4.8) | 102 (4.3) | 1.12 (0.79, 1.60) | 0.531 |
| Composite valve complication$ | - | - | - | - |  | 226 (24.9) | 679 (28.9) | 0.84 (0.72, 0.98) | 0.022 |
| Redo valve surgery | - | - | - | - |  | 87 (9.6) | 178 (7.6) | 1.29 (1.01, 1.67) | 0.052 |
| MACCEs# | - | - | - | - |  | 393 (43.3) | 1,052 (44.7) | 0.96 (0.86, 1.08) | 0.528 |

Abbreviation: IE, infective endocarditis; HR, hazard ratio; CI, confidence interval; IE, infective endocarditis; ESRD, end-stage renal disease; MACCEs, major adverse cardiac and cerebrovascular events.

$ Anyone of major bleeding, stroke or readmission due to infective endocarditis.

# Anyone of all-cause death, stroke, hospitalization for heart failure or redo valve surgery.

Data were presented as frequency (percentage).

**Supplementary Table 6**. In-hospital outcomes of female and male patients with IE in the whole cohort and in the subgroup undergoing valve surgery after matching, without considering urbanization level of the residence and hospital level of the index IE hospitalization in propensity score calculation

|  | Total | | | |  | Valve surgery | | | |
| --- | --- | --- | --- | --- | --- | --- | --- | --- | --- |
| Variable | Women  (*n* = 9,873) | Men  (*n* = 9,873) | OR/B (95% CI) | *P* value |  | Women  (*n* = 1,219) | Men  (*n* = 1,219) | OR/B (95% CI) | *P* value |
| In-hospital death | 2,106 (21.3) | 1,974 (20.0) | 1.09 (1.01, 1.16) | 0.020 |  | 249 (20.4) | 168 (13.8) | 1.61 (1.30, 1.99) | <0.001 |
| 30-day mortality | 1,498 (15.2) | 1,412 (14.3) | 1.07 (0.99, 1.16) | 0.084 |  | 128 (10.5) | 78 (6.4) | 1.72 (1.28, 2.30) | <0.001 |
| Cardiogenic shock requiring MCS | 166 (1.7) | 144 (1.5) | 1.16 (0.92, 1.45) | 0.208 |  | 129 (10.6) | 89 (7.3) | 1.50 (1.13, 1.99) | 0.005 |
| Re-exploration for bleeding | 36 (0.4) | 44 (0.4) | 0.82 (0.53, 1.27) | 0.371 |  | 30 (2.5) | 32 (2.6) | 0.94 (0.57, 1.55) | 0.797 |
| Newly-onset stroke | 924 (9.4) | 898 (9.1) | 1.03 (0.94, 1.14) | 0.523 |  | 179 (14.7) | 181 (14.8) | 0.99 (0.79, 1.24) | 0.909 |
| De novo dialysis | 579 (5.9) | 522 (5.3) | 1.12 (0.99, 1.26) | 0.077 |  | 168 (13.8) | 120 (9.8) | 1.46 (1.14, 1.88) | 0.003 |
| Tracheostomy | 262 (2.7) | 244 (2.5) | 1.08 (0.90, 1.28) | 0.418 |  | 70 (5.7) | 58 (4.8) | 1.22 (0.85, 1.74) | 0.277 |
| Hospital stay, days | 32.5 ± 28.6 | 31.6 ± 26.5 | 0.87 (0.10, 1.63) | 0.027 |  | 54.0 ± 32.8 | 50.9 ± 27.3 | 3.09 (0.69, 5.49) | 0.012 |
| Ventilator use, days | 4.25 ± 11.17 | 3.84 ± 10.25 | 0.42 (0.12, 0.72) | 0.006 |  | 10.33 ± 14.82 | 8.14 ± 12.74 | 2.18 (1.09, 3.28) | <0.001 |

Abbreviation: IE, infective endocarditis; OR, odds ratio; *B*, unstandardized regression coefficient; CI, confidence interval; MCS, mechanical circulation support;

Data were presented as frequency (percentage) or mean ± standard deviation.

**Supplementary Table 7**. Late outcomes of female and male patients with IE in the whole cohort and in the subgroup undergoing valve surgery after matching, without considering urbanization level of the residence and hospital level of the index IE hospitalization in propensity score calculation

|  | Total | | | |  | Valve surgery | | | |
| --- | --- | --- | --- | --- | --- | --- | --- | --- | --- |
| Outcome | Women  (*n* = 7,767) | Men  (*n* = 7,899) | HR (95% CI) | *P* value |  | Women  (*n* = 970) | Men  (*n* = 1,051) | HR (95% CI) | *P* value |
| Hospitalization for heart failure | 701 (9.0) | 689 (8.7) | 0.995 (0.90, 1.10) | 0.923 |  | 91 (9.4) | 87 (8.3) | 1.10 (0.83, 1.47) | 0.492 |
| Stroke | 749 (9.6) | 834 (10.6) | 0.87 (0.79, 0.96) | 0.005 |  | 96 (9.9) | 132 (12.6) | 0.75 (0.57, 0.99) | 0.041 |
| Readmission due to IE | 1,265 (16.3) | 1,483 (18.8) | 0.83 (0.77, 0.90) | <0.001 |  | 165 (17.0) | 192 (18.3) | 0.93 (0.76, 1.14) | 0.470 |
| Readmission due to any cause | 6,266 (80.7) | 6,382 (80.8) | 0.98 (0.95, 1.02) | 0.347 |  | 719 (74.1) | 796 (75.7) | 0.997 (0.90, 1.10) | 0.945 |
| ESRD requiring permanent dialysis | 277 (3.6) | 240 (3.0) | 1.13 (0.95, 1.34) | 0.175 |  | 28 (2.9) | 28 (2.7) | 1.05 (0.62, 1.78) | 0.862 |
| Major bleeding | 715 (9.2) | 880 (11.1) | 0.78 (0.71, 0.86) | <0.001 |  | 89 (9.2) | 121 (11.5) | 0.76 (0.58, 0.99) | 0.041 |
| Pacemaker implantation | 270 (3.5) | 254 (3.2) | 1.03 (0.87, 1.22) | 0.719 |  | 54 (5.6) | 54 (5.1) | 1.05 (0.72, 1.54) | 0.798 |
| Composite valve complication$ | - | - | - | - |  | 284 (29.3) | 333 (31.7) | 0.90 (0.76, 1.05) | 0.170 |
| Redo valve surgery | - | - | - | - |  | 112 (11.5) | 80 (7.6) | 1.53 (1.16, 2.03) | 0.003 |
| MACCEs# | - | - | - | - |  | 482 (49.7) | 543 (51.7) | 0.96 (0.85, 1.08) | 0.458 |

Abbreviation: IE, infective endocarditis; HR, hazard ratio; CI, confidence interval; IE, infective endocarditis; ESRD, end-stage renal disease; MACCEs, major adverse cardiac and cerebrovascular events;

$ Anyone of major bleeding, stroke or readmission due to infective endocarditis;

# Anyone of all-cause death, stroke, hospitalization for heart failure or redo valve surgery;

Data were presented as frequency (percentage).
