# Supplementary figures and images for: Sex Differences in Clinical Outcomes and Surgical Interventions for Infective Endocarditis: A Nationwide Registry
Source: Open Forum Infect Dis. 2025 Aug 12;12(8):ofaf473. doi: 10.1093/ofid/ofaf473 (PMC12372668; doi:10.1093/ofid/ofaf473)

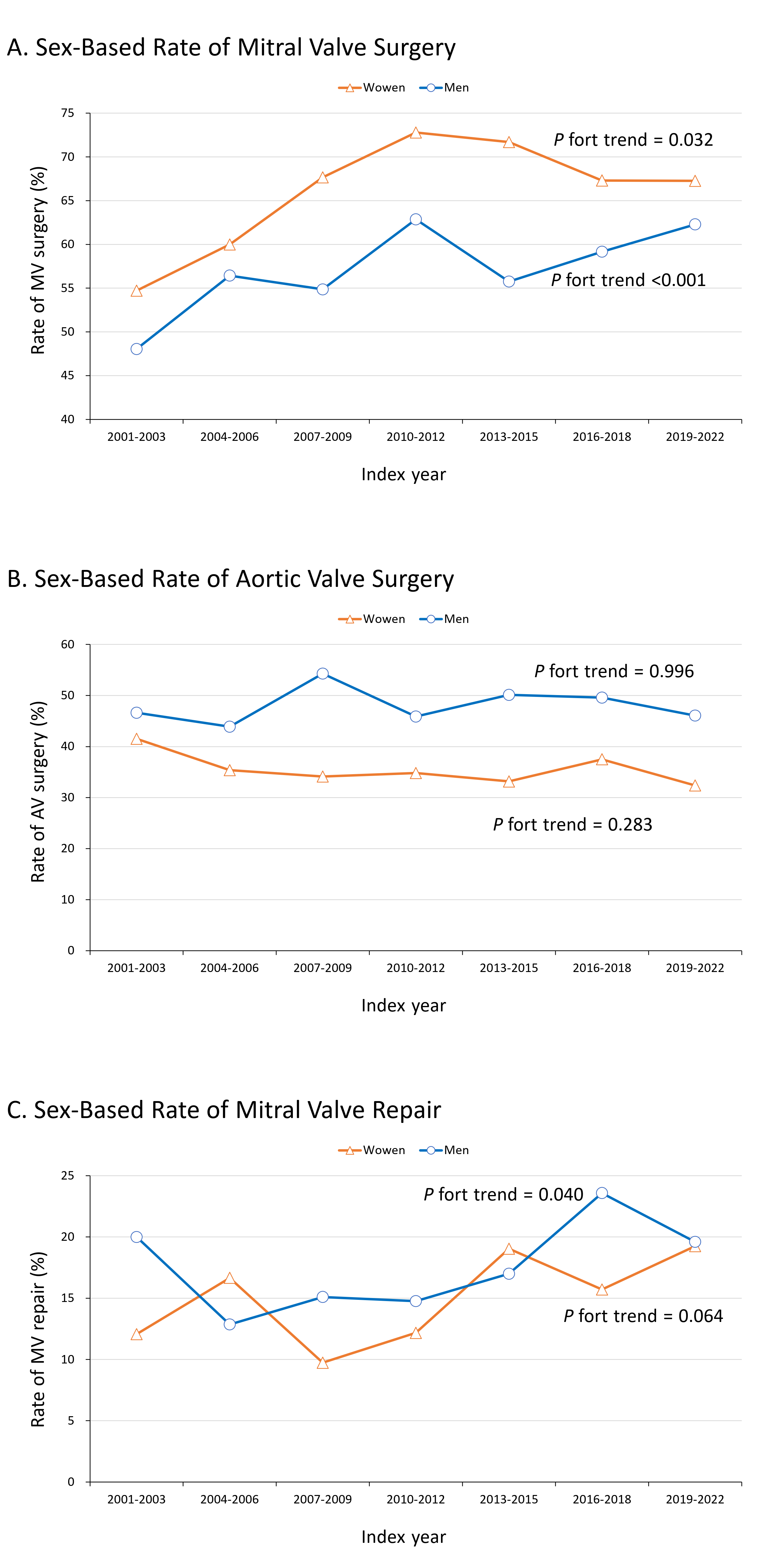

Supplement: ofaf473_Supplementary_Data [file ofaf473_supplementary_data.zip › Figure S2ABC.tif]

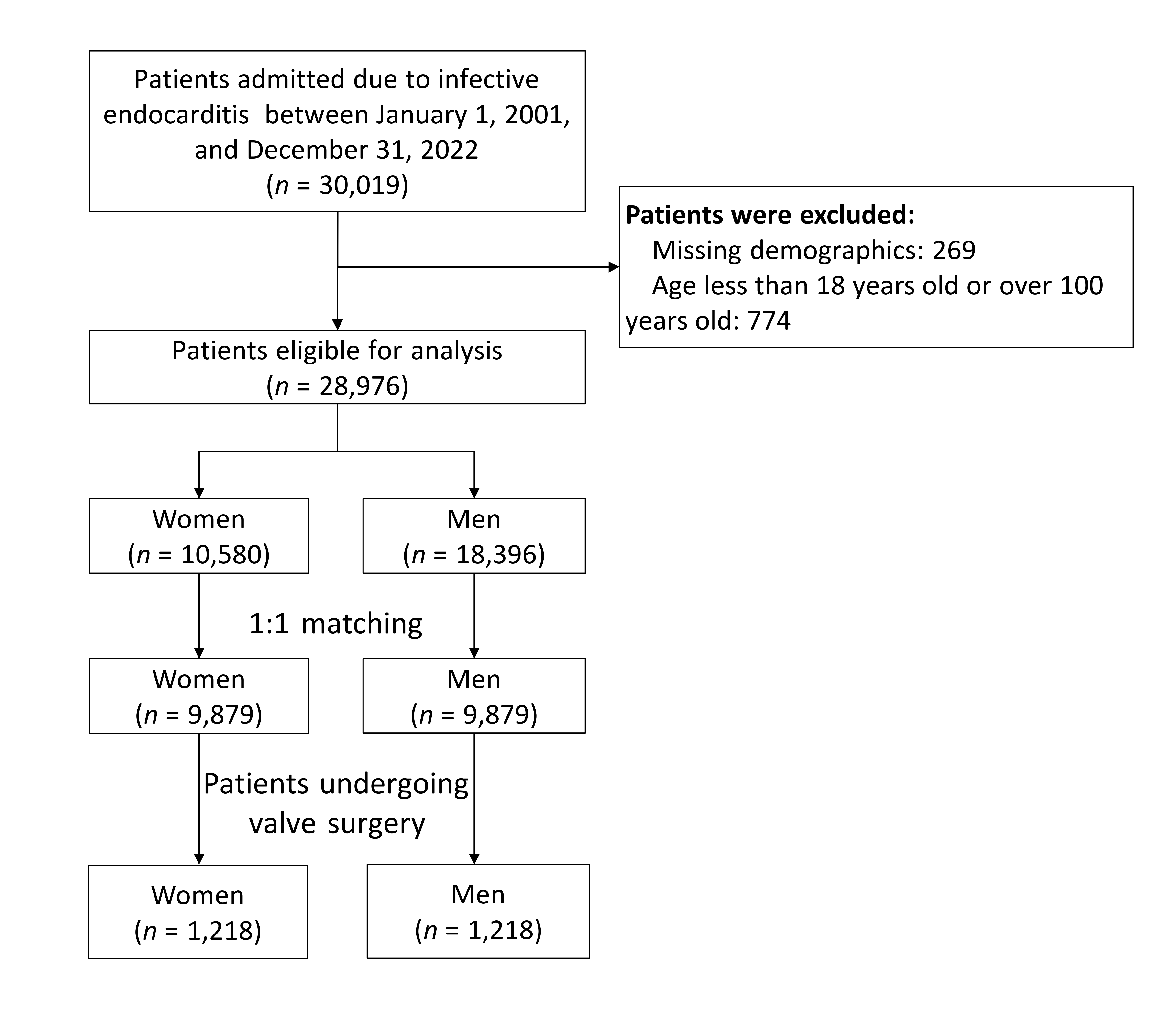

Supplement: ofaf473_Supplementary_Data [file ofaf473_supplementary_data.zip › Figure S1.tif]
